# Mutation of SLC35D3 Causes Metabolic Syndrome by Impairing Dopamine Signaling in Striatal D1 Neurons

**DOI:** 10.1371/journal.pgen.1004124

**Published:** 2014-02-13

**Authors:** Zhe Zhang, Chan-Juan Hao, Chang-Gui Li, Dong-Jie Zang, Jing Zhao, Xiao-Nan Li, Ai-Hua Wei, Zong-Bo Wei, Lin Yang, Xin He, Xue-Chu Zhen, Xiang Gao, John R. Speakman, Wei Li

**Affiliations:** 1 State Key Laboratory of Molecular Developmental Biology, Institute of Genetics and Developmental Biology, Chinese Academy of Sciences, Beijing, China; 2 Graduate School of Chinese Academy of Sciences, Beijing, China; 3 Reproductive and Genetic Center, National Research Institute for Family Planning, Beijing, China; 4 The Affiliated Hospital of Qingdao University Medical College, Qingdao, China; 5 Model Animal Research Center, Nanjing University, Nanjing, China; 6 Nanjing Children's Hospital, Nanjing Medical University, Nanjing, China; 7 Beijing Tongren Hospital, Capital Medical University, Beijing, China; 8 Department of Pharmacology, Soochow University College of Pharmaceutical Sciences, Suzhou, China; 9 Institute of Biological and Environmental Sciences, University of Aberdeen, Aberdeen, Scotland, United Kingdom; Yale University, United States of America

## Abstract

Obesity is one of the largest health problems facing the world today. Although twin and family studies suggest about two-thirds of obesity is caused by genetic factors, only a small fraction of this variance has been unraveled. There are still large numbers of genes to be identified that cause variations in body fatness and the associated diseases encompassed in the metabolic syndrome (MetS). A locus near a sequence tagged site (STS) marker D6S1009 has been linked to obesity or body mass index (BMI). However, its genetic entity is unknown. D6S1009 is located in the intergenic region between *SLC35D3* and *NHEG1*. Here we report that the *ros* mutant mice harboring a recessive mutation in the *Slc35d3* gene show obesity and MetS and reduced membrane dopamine receptor D1 (D1R) with impaired dopamine signaling in striatal neurons. SLC35D3 is localized to both endoplasmic reticulum (ER) and early endosomes and interacts with D1R. In *ros* striatal D1 neurons, lack of SLC35D3 causes the accumulation of D1R on the ER to impair its ER exit. The MetS phenotype is reversible by the administration of D1R agonist to the *ros* mutant. In addition, we identified two mutations in the *SLC35D3* gene in patients with MetS, which alter the subcellular localization of SLC35D3. Our results suggest that the *SLC35D3* gene, close to the D6S1009 locus, is a candidate gene for MetS, which is involved in metabolic control in the central nervous system by regulating dopamine signaling.

## Author Summary

Genome-wide linkage analyses have revealed that an STS marker D6S1009 (about 55 kb from the *SLC35D3* gene) is linked to obesity or BMI in the Framingham Heart Study, but its genetic entity is unknown. Here we characterized the features of obesity and metabolic syndrome with reduced physical activity levels in a previously identified *ros* mouse mutant, carrying a homozygous *Slc35d3* mutation. These *ros* phenotypes were caused by the intracellular accumulation of D1R mostly on ER in the striatal neurons, impairing D1R signaling and reducing energy expenditure. In addition, we identified two mutations of *SLC35D3* in patients with metabolic syndrome which are subcellularly mislocalized. We propose that the *SLC35D3* gene is likely a novel candidate gene for MetS and obesity.

## Introduction

The worldwide prevalence of obesity (OMIM 601665, http://www.ncbi.nlm.nih.gov/omim/) is increasing (data from the International Obesity Taskforce (IOTF) website, http://www.iaso.org/iotf/obesity/). This has resulted in a significant increase in morbidity and mortality associated with the metabolic syndrome (MetS, OMIM 605552). Obesity and associated MetS or body mass index (BMI, OMIM 606641) are regarded as complex traits influenced by both additive genetic effects and environmental factors [1]. It has been estimated that genetic factors explain 67% of the variance in human obesity [2]. Currently, more than 150 loci have been implicated in the development of monogenic obesity, syndromic obesity and polygenic obesity. However, only about 2% of the variance in this trait has been explained [3], [4]. About 200 cases of severe obesity have been reported to be associated with a single gene mutations in a cohort of 11 genes [5]. Studies on extremely obese children have been successful in the characterization of the causative genes for monogenic obesity. However, progress with this approach has been very slow, and is expected to be faster in the era of whole exome sequencing. On the other hand, the identification of the *FTO* gene as an obesity gene is an example of loci uncovered by genome-wide association or linkage studies [6]. It remains a challenge to uncover genes responsible for mild or moderate obese phenotypes, especially those which develop in adulthood.

Genome-wide linkage analyses have revealed that a locus on chromosome 6q23-25 is linked to obesity in the Framingham Heart Study, with a major locus near the sequence tagged site (STS) marker D6S1009 [7]–[9]. D6S1009 is located within the intergenic region between *SLC35D3* (55,419 bp apart at the centromere side) and *NHEG1* (867 bp apart at the telomere side) in the NCBI Map Viewer (http://www.ncbi.nlm.nih.gov/mapview/). *NHEG1* (neuroblastoma highly expressed 1) is a predicted gene with unknown function. No association with obesity of this gene has been documented. SLC35D3 (solute carrier family 35, member D3) is predicted as an orphan nucleotide sugar transporter or a fringe connection-like protein with 10 transmembrane domains. Previous studies have characterized the recessively inherited *ros-/−* mutant mouse (*ros* hereafter), which has a spontaneous intracisternal A particle (IAP) insertion at the first exon of the *Slc35d3* gene to disrupt its function [10]. Platelet dense granules are absent in the *ros* mutant, suggesting that SLC35D3 is involved in the biogenesis of platelet dense granules [10], [11]. This function seems not to do with the solute carrier, and requires further investigation. Mouse *Slc35d3* is specifically expressed in the brain as determined by multiple tissue Northern blots [10], suggesting it has specific roles in the central nervous system. In addition, *Slc35d3* expression was restricted to the striatonigral medium spiny neurons (MSNs) expressing dopamine receptor D1 (D1R) rather than the striatopallidal MSNs expressing dopamine receptor D2 (D2R) [12]. Interestingly, during the breeding of this mouse, we observed that the adult *ros* mice gained weight progressively. Here we have characterized the *ros* mutant as a mouse model of MetS and obesity. In addition, we found two MetS patients with mutations of the *SLC35D3* gene. Our results suggest that *SLC35D3* is a candidate gene for obesity-related MetS, which is involved in metabolic control in the central nervous system by regulating dopamine signaling.

## Results

### *Ros* mutant exhibits metabolic syndrome and lowered energy expenditure

**Figure 1:**
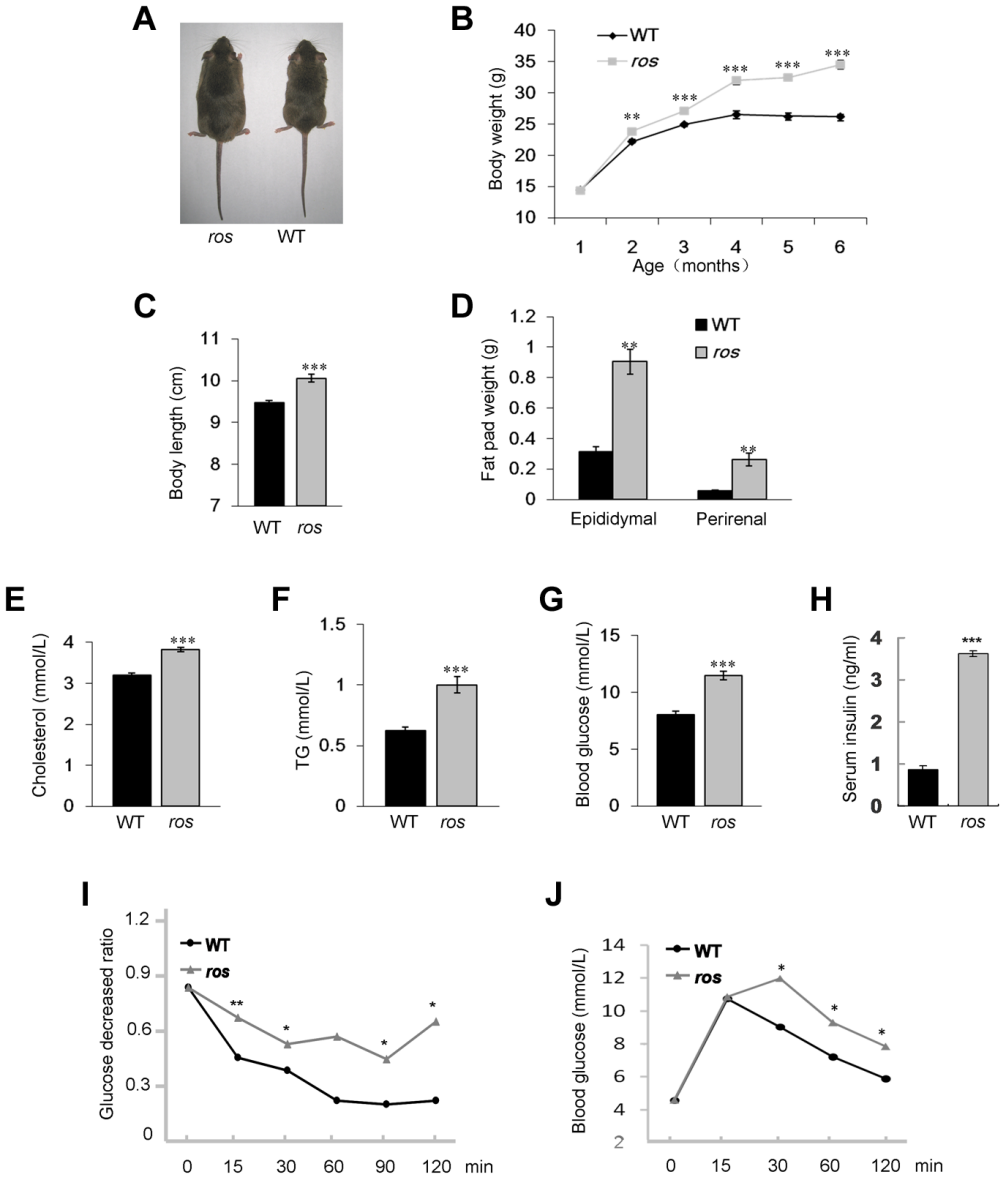
*Ros* mice exhibit features of metabolic syndrome. (**A**) Comparison of *ros* mouse and wild-type (WT) C3H/HeSnJ mouse at 24 weeks of age. (**B**) Growth curves of male WT and *ros* mice. Body weights of *ros* mice are significantly higher than those of WT starting at 8 weeks. (**C**) *ros* mice show significantly increased naso-to-anal body length (10.06±0.09 cm, n = 10) compared with the WT controls (9.47±0.06 cm, n = 10) at 24 weeks of age. (**D**) Perirenal and epididymal fat pad weight in *ros* mice (n = 10) are significantly higher than those of the WT (n = 10) respectively at 24 weeks of age. (**E–G**) Serum levels of cholesterol (Chol), triglycerides (TG) and blood glucose (Gluc) in fasted *ros* mice (n = 14) are significantly higher than those in WT controls (n = 14) at 24 weeks of age. In *ros*, mean values of Chol, TG, Gluc are 3.82, 1.0, 11.47 mmol/L, respectively; In WT, mean values of Chol, TG, Gluc are 3.21, 0.62, 8.03 mmol/L, respectively. (**H**) Plasma insulin levels were increased in non-fasted *ros* mice (3.62±0.20 ng/ml, n = 3) than in WT (0.87±0.08 ng/ml, n = 3) at 24 weeks of age. (**I**) Insulin tolerance tests (ITT) showed a tendency of impaired tolerance in *ros* mice compared with WT mice at 24 weeks of age (n = 3). (**J**) Glucose tolerance tests (ITT) showed a tendency of impaired tolerance in *ros* mice compared with WT mice at 24 weeks of age (n = 3). *P<0.05, **P<0.01, ***P<0.001.

During the breeding of the *ros* mutant mice, we observed that adult *ros* mice became obese compared to sex and age-matched wild-type (WT) mice (Fig. 1A). Growth curves showed progressive and significant weight gain of *ros* mice relative to WT controls, starting at 8 weeks in males, which is similar to the features of late-onset obesity in humans. However, the expression levels of SLC35D3 in the striatum did not change at different postnatal stages within 6 months (Fig. S1). Based on this observation, we chose mice at 12 weeks of age for behavior and molecular tests, mice at 24 weeks of age for phenotypic analyses of MetS. At 24 weeks of age, *ros* males were 31.5% heavier than age-matched WT males (Fig. 1B), while the naso-to-anal body length was increased by 6.2% in *ros* mice relative to WT controls (Fig. 1C).

To determine whether the increased weight of *ros* mice reflects body composition changes, we dissected and weighed two distinct fat pads, epididymal and perirenal white adipose tissue (WAT). Both epididymal and perirenal fat mass were enormously increased in *ros* mice (Fig. 1D). Serum cholesterol and triglycerides levels were increased about 19% and 61% respectively compared with the WT controls (Figs. 1E and 1F). Blood glucose levels in *ros* mice were increased about 43% (Fig. 1G). In addition, serum insulin was increased about 4.2-fold in *ros* mice (Fig. 1H), while the insulin tolerance test (ITT) and glucose tolerance test (GTT) showed *ros* mice were insulin resistant and glucose intolerant (Figs. 1I and 1J). Taken together, the *ros* mice exhibited multiple features of the MetS with late-onset obesity, hyperlipidemia, hyperglycemia and hyperinsulinemia.

**Figure 2:**
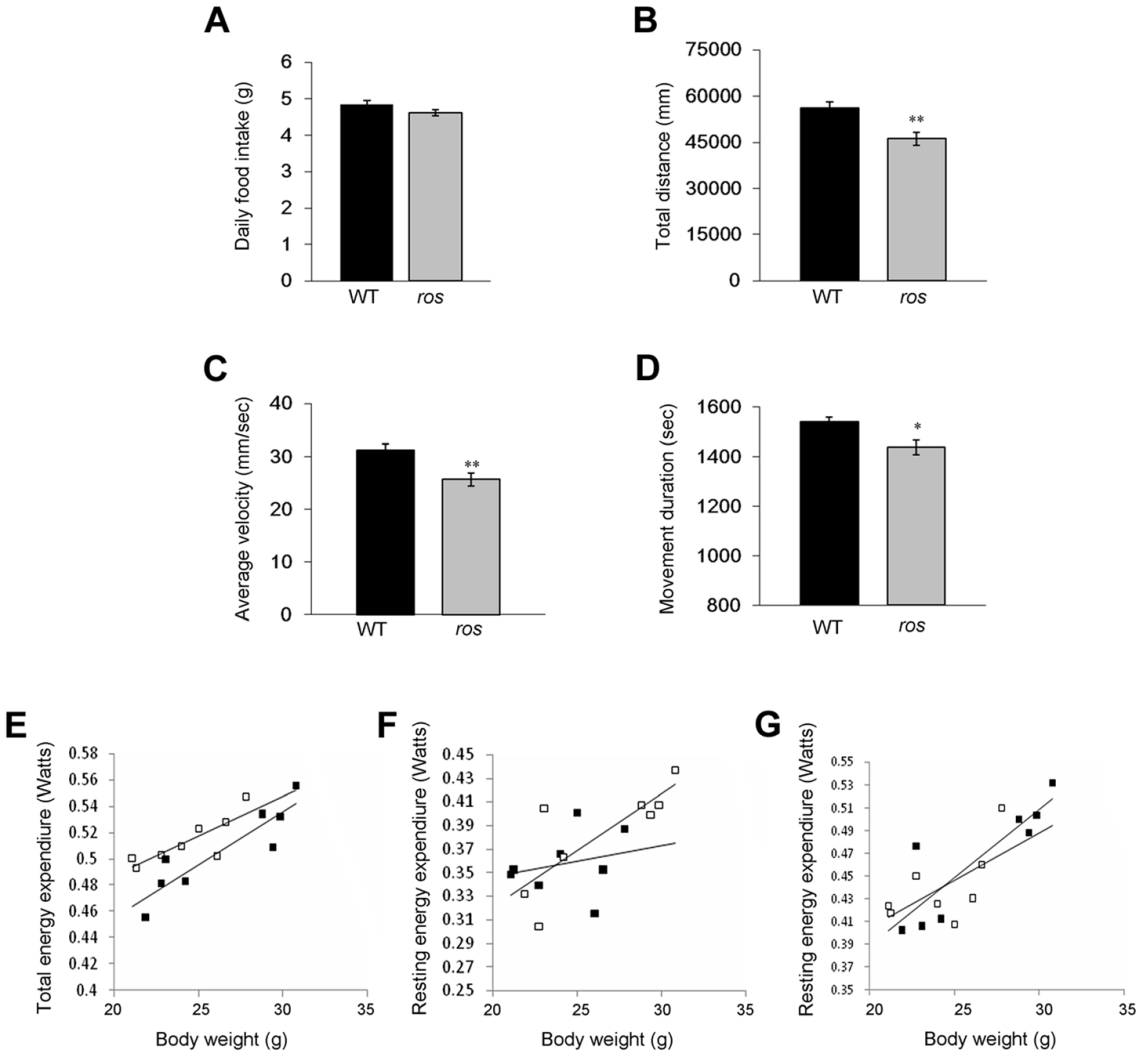
Food intake, spontaneous locomotor activity and total and resting energy expenditure. Mice at 12 weeks of age were used in this study. (**A**) There was no significant difference in daily food intake between wild-type (WT) (n = 14) and *ros* mice (n = 15). *P* = 0.066. (**B**) The distance traveled during 30 min for *ros* mice (n = 25) was lower than that for the WT (n = 20). (**C**) The average movement velocity during 30 min for *ros* mice (n = 25) was shorter than the WT (n = 20). (**D**) The total movement duration during 30 min for *ros* mice (n = 25) was shorter than the WT (n = 20). (**E–G**) Total energy expenditure (Watts) (E), resting energy expenditure (Watts) by Method 1 (F) and resting energy expenditure (Watts) by Method 2 (G) for WT and *ros* mice measured at age 8 to 12 weeks (n = 8 per group). WT mice are black symbols and *ros* mice are white symbols. Fitted linear regressions for each data set are shown. There was a significant effect of body weight and genotype but no interaction in total energy expenditure (ANCOVA: Body weight effect, F_1,13_ = 44.85, p<0.001); genotype effect, F_1,13_ = 10.47, p = 0.007; interaction, not significant (ns).). There was a significant effect of body weight but no genotype or interaction effect in resting energy expenditure by Method 1 or 2 (Method 1 ANCOVA: Body weight effect, F_1,13_ = 8.78, p = .011; genotype effect, F_1,13_ = 0.35, p = 0.566; interaction: ns. Method 2 ANCOVA: Body weight effect, F_1,13_ = 22.29, p<0.001; genotype effect, F_1,13_ = 0.03, p = 0.873; interaction, ns.). *P<0.05, **P<0.01.

The development of obese *ros* mice may result from elevated energy intake and/or decreased energy expenditure. To assess whether *ros* mice were hyperphagic, daily food intake was monitored in animals fed a standard chow diet *ad libitum* for 7 consecutive days at the age of 24 weeks when *ros* mice were already obese. There was no significant difference in daily food intake between WT and *ros* mice (Fig. 2A). This suggests that energy intake is unaffected in *ros* mice. On the other hand, *ros* mice had significantly decreased physical activity including decreased movement distance, average velocity and movement duration (Figs. 2B–D).

The decreased physical activity in *ros* mice prompted us to measure daily energy expenditure over 5 day periods using an Oxymax system (details in the Materials and Method and Fig. S2). Total energy expenditure (resting plus activity metabolism) in the *ros* mice at age 2–3 months, prior to development of obesity, was significantly lower than in the WT mice using analysis of covariance (ANCOVA) [13] (Fig. 2E). However, there was no difference between the genotypes in their resting metabolic rates, independent of how the resting metabolism was evaluated (Figs. 2F and 2G). This indicated that the difference in energy expenditure between the genotypes was contributed to only by the differences in physical activity expenditure. When we included distance traveled in the respirometry chambers as a covariate in the analysis of total metabolic rate, this only marginally reduced the effect of genotype (F_1,12_ = 11.48, *P* = 0.05), suggesting the impact of the mutation is on the total amount of activity as well as the energy costs of locomotion.

### Plasma membrane D1R is reduced in *ros* striatonigral neurons which impairs dopamine signaling

**Figure 3:**
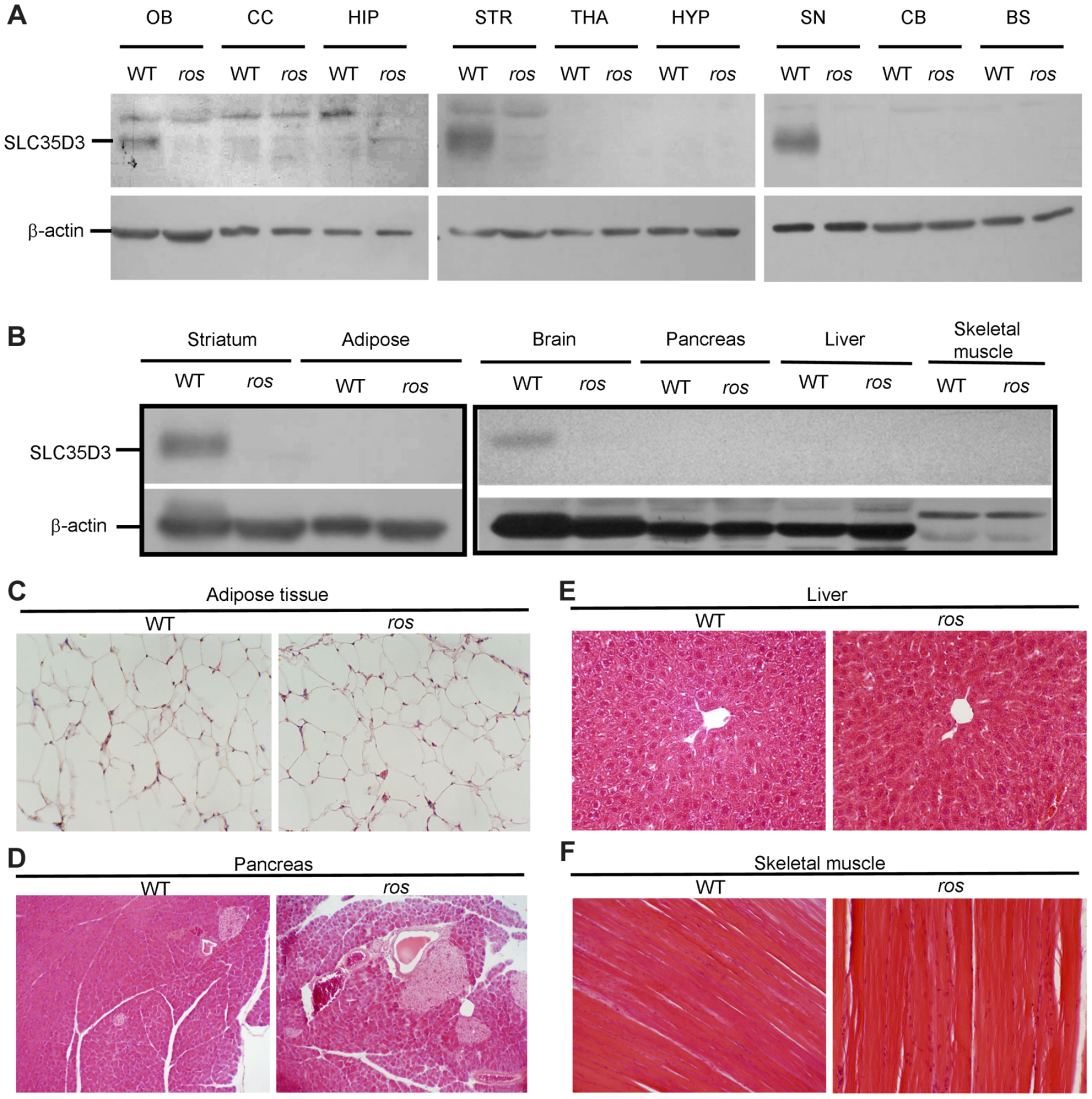
SLC35D3 expression in brain sub-regions and non-neuronal tissues. Mice at 24 weeks of age were used in this study. (**A**) Immunoblotting of SLC35D3 protein in nine brain sub-regions of wild-type (WT) and *ros* mice. OB: olfactory bulb, CC: cerebral cortex, HIP: hippocampus, STR: striatum, THA: thalamus, HYP: hypothalamus, SN: substantia nigra, CB: cerebellum, BS: brain stem. (**B**) Immunoblotting assay showed no expression of SLC35D3 in adipose tissue, pancreas, liver, or skeletal muscle compared with striatum or brain in WT mice. There was no expression of SLC35D3 in any of these tissues in *ros* mice. β-actin serves as a loading control. (**C**) The size of adipose from epididymal fat pad of *ros* mice was normal. (**D–F**) No apparent fat accumulation was observed in pancreas, liver, or skeletal muscle in *ros* mice. 200× magnification was applied in the sections with H–E staining.

The brain-specific expression pattern in the multiple Northern blots [10] indicated a restricted expression and specific function of SLC35D3 in the brain. We detected no mutant SLC35D3 protein in striatum, substantia nigra and olfactory bulb of the *ros* mutant with an antibody to mouse SLC35D3 (Fig. 3A), although transcription was upregulated in mutant tissues [10]. SLC35D3 protein was readily detectable in these tissues of WT mice (Fig. 3A). Although the expression of SLC35D3 in the Allen Brain Atlas (http://www.brain-map.org/) shows a wider distribution, in either WT or mutant mice, we did not detect the SLC35D3 protein in other brain sub-regions and especially in the obesity-related brain tissues such as thalamus and hypothalamus (Fig. 3A), as well as in several organs involved in energy homeostasis such as adipose tissue, pancreas, liver and skeletal muscle (Fig. 3B). In addition, no apparent morphological changes or fat accumulation was observed in these organs (Figs. 3C–3F). Considering the enlargement of WAT fat pads (Fig. 1D), the adipocytes in adult *ros* mice exhibited hyperplasia (increase in number), rather than hypertrophy (increase in size), to contribute mainly to the weight gain. The presence of SLC35D3 in non-neuronal tissues is only known so far in platelets and it plays a role in the biogenesis of platelet dense granules [10], [11]. These results suggest that SLC35D3 is selectively expressed in certain types of neurons with particular enrichment in the basal ganglia, and that *ros* mice allow us to investigate the phenotypes related to its dysfunction in these neurons.

**Figure 4:**
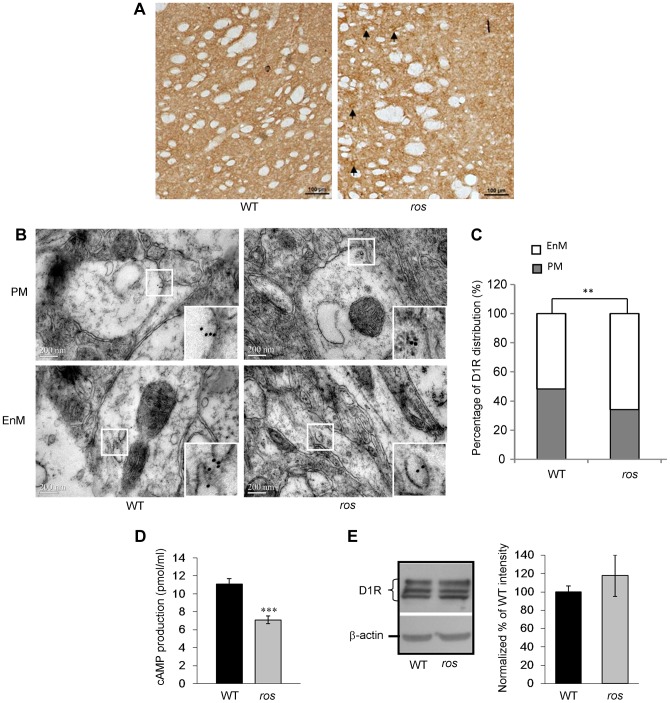
Plasma membrane D1R and its signaling are reduced in *ros* striatonigral neurons. Mice at 12 weeks of age were used in this study. (**A**) Immunohistochemical detection of D1R on the coronal brain sections of the striatum. In *ros* mice, visible immunoreactive cell bodies are present (arrows). Scale bar: 100 µm. (**B, C**) Representative immuno-EM pictures of D1R-labeled particles on plasma membrane (PM, upper panels) and endomembrane structures (EnM, lower panels) in wild-type (WT) and *ros* striatal neurons are shown without much difference (B). However, the quantification test revealed that the particles on PM in *ros* mice (34.0%, n = 103) is significantly lower than that in WT (51.7%, n = 87), **P<0.01. Scale bar: 200 nm. (**D**) SKF82958–induced cAMP accumulation in striatal membranes prepared from WT and *ros* mice. As compared with the WT (11.09±0.61 pmol/ml, n = 6), cAMP activity in *ros* membranes (7.11±0.45 pmol/ml, n = 6) was reduced about 36%. *P<0.001. (**E**) Immunoblot analysis of D1 receptor. *left panel*, Striatum tissues (20 µg) from WT and *ros* mice were probed with the monoclonal D1R antibody. β-actin was used as a loading control. The immunoblots shown are representative of three independent experiments. *right panel*, Normalized percentages of the band intensities shown in *left panel* are means ± SEM (n = 3). There is no significant difference of total D1R levels between WT and *ros* mice (*P*>0.05).

It has been reported that *Slc35d3* is specifically expressed in the striatonigral MSNs expressing D1R rather than the striatopallidal MSNs expressing D2R [12], therefore, we investigated whether SLC35D3 regulates the function of D1 neurons. Immunohistochemical analysis using an antibody to D1R showed that numerous intensely immunoreactive cell bodies were present in *ros* striatum (Fig. 4A), which is similar to the intracellular accumulation or internalization of D1R after D1R agonist treatment [14]. To confirm this, in immuno-electronic microscopic (IEM) pictures labeled by anti-D1R, we quantified the gold-labeled particles located on the plasma membrane (PM) and endomembrane structures (EnM) (Fig. 4B). The proportion of D1R in EnM was significantly higher (66%) in *ros* striatum than that (48.3%) in wild-type (Fig. 4C).

We then tested cyclic AMP (cAMP) production to detect functional D1R activation at the cell surface. Stimulation of D1R by the specific agonist SKF82958 (10 µM) produced an accumulation of cAMP in both groups. Consistently, cAMP production in *ros* striatum was reduced about 36% compared with the WT controls (Fig. 4D), which may be attributable to the reduction of plasma membrane D1R. Western blotting of striatum lysates showed that total D1 receptor expression levels were similar between wild-type and *ros* samples (Fig. 4E), indicating the total number of striatal D1 neurons in *ros* mice is not changed. In comparison, total D2R and the fraction of D2R on the plasma membrane were unchanged in the striatum of *ros* mutant mice (Fig. S3), consistent with the observation that SLC35D3 is not expressed in the D2 neurons [12]. This indicates that SLC35D3 plays a role in D1R trafficking in the striatal D1 neurons, but does not affect D2R trafficking in the striatal D2 neurons. Taken together, our results suggest that loss of SLC35D3 in *ros* striatum causes intracellular accumulation of D1R and reduces D1 receptors on the plasma membrane.

### SLC35D3 is localized to the ER and early endosomes and it interacts with D1R

**Figure 5:**
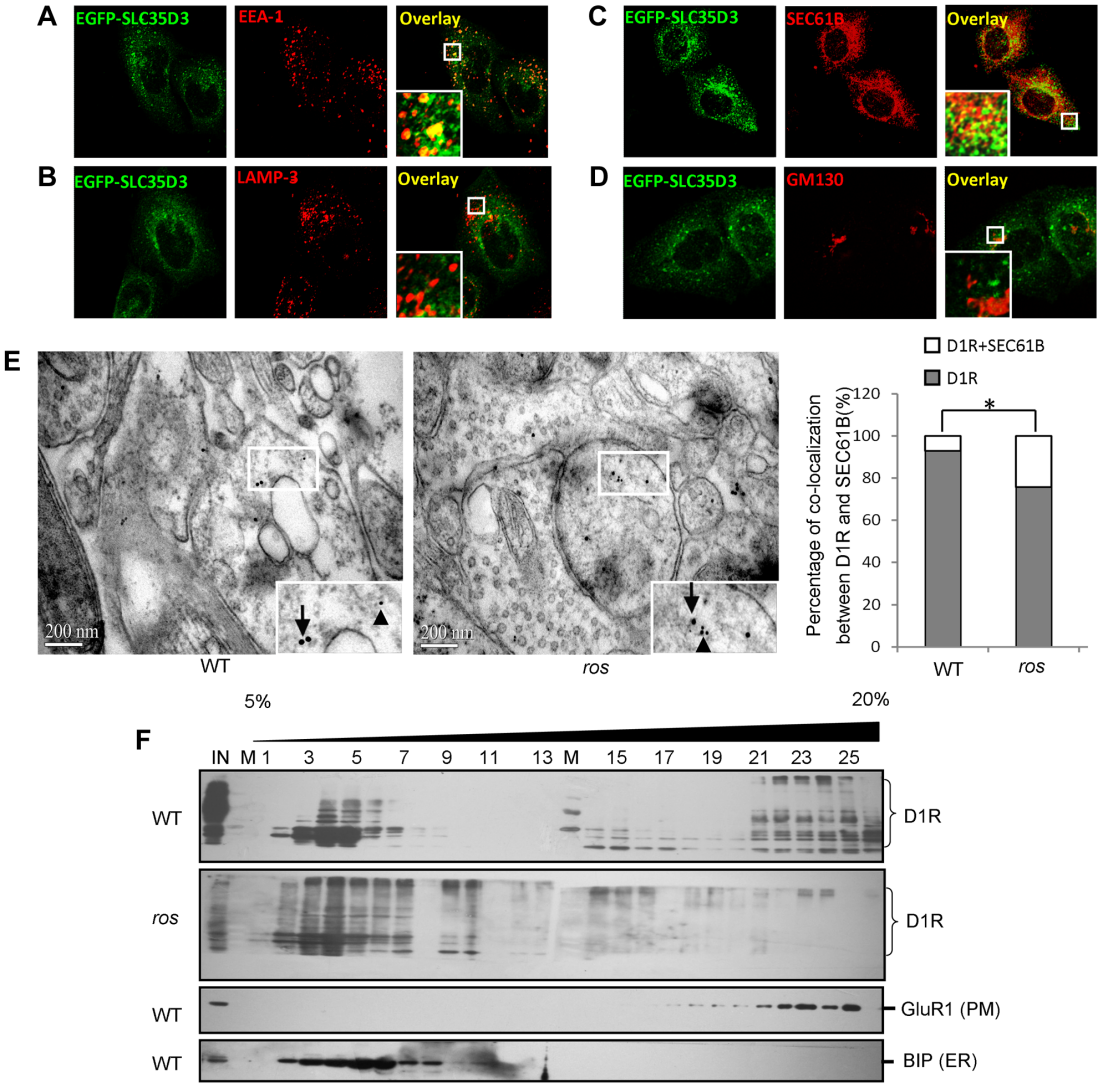
Subcellular localization of SLC35D3 protein and the effect of SLC35D3 on trafficking of D1R. (**A–D**) In HEK293T cells, EGFP-SLC35D3 co-localized with the ER marker SEC61B and the endosome marker EEA1, but not with the Golgi marker GM130 or the lysosome/late endosome marker LAMP3. (**E**) Representative pictures to show that **t**he smaller gold-labeled D1R particles (10 nm, arrowheads) and the larger gold-labeled SEC61B particles (ER marker) (15 nm, arrows) are colocalized in *ros* striatum compared with the particles on different structures in wild-type (WT) under immuno-EM at 12 weeks of age. Scale bar: 200 nm. The percentage of D1R colocalized with SEC61B in *ros* mutant (19.6%, n = 97) is significantly higher than that in wild-type (WT) (6.8%, n = 74), *P<0.05. (**F**) Immunoblotting of Optiprep (Biocomp, USA) gradient (5–20%) fractions. One microgram tissue lysates of striatum at 12 weeks of age were separated into 26 fractions. D1R was shifted from plasma membrane (PM: GluR1 as a marker) in fractions 16–25 to intracellular fractions 2–10 mainly in endoplasmic reticulum (ER: BIP as a marker) in *ros* mice compared with WT mice. IN: lysate input; M: molecular marker.

To ascertain the underlying mechanism of the accumulation of D1R within the *ros* neurons, we first examined the subcellular localization of mouse SLC35D3. Consistent with a recent report [11], the EGFP-SLC35D3 protein was selectively localized to the ER and early endosomes, but not to the Golgi apparatus or late endosomes/lysosomes (Figs. 5A–5D).

We then investigated the intracellular location of accumulated D1R in *ros* striatal neurons. We performed immuno-EM by double-labeling with anti-D1R and anti-SEC61B (an ER marker) and found that the D1R particles co-residing with SEC61B in *ros* striatal neurons (19.6%) were significantly higher than that in wild-type (6.8%) (Fig. 5E). This indicates that the increased proportion of ER-retained D1R (12.8%, Fig. 5E) may mostly account for the reduction of plasma membrane D1R (17.7%, Fig. 4B) in *ros* mice. Our OptiPrep gradient assays further confirmed the shift of D1R from plasma membrane (fractions 16–25) to intracellular fractions 2–10 mainly corresponding to ER in *ros* striatum compared with the wild-type (Fig. 5F).

**Figure 6:**
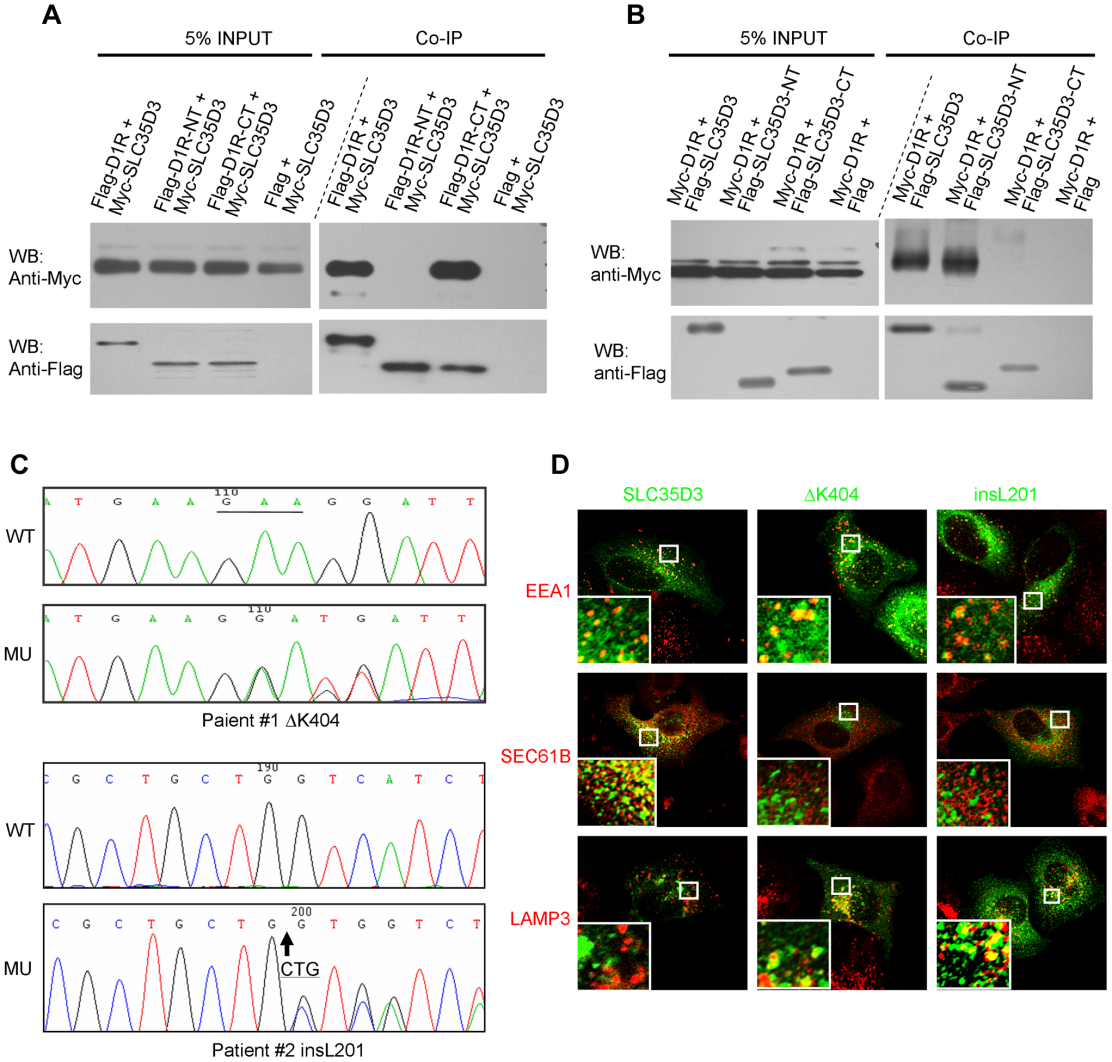
SLC35D3 interacts with D1R and mutations of *SLC35D3* alter its subcellular distribution. (**A**) C-terminal D1R interacted with SLC35D3. Cell lysates with the co-expression of Myc-SLC35D3 and Flag-D1R (or Flag-D1R-NT (1–216aa), or Flag-D1R-CT (217–446aa)) were immunoprecipitated. Both the full-length D1R and the D1R-CT bound with Myc-SLC35D3. (**B**) N-terminal SLC35D3 interacted with D1R. Cell lysates with the co-expression of Myc-D1R and Flag-SLC35D3 (or Flag-SLC35D3-NT (1–301aa), or Flag-SLC35D3-CT (242–422aa)) were immunoprecipitated. Both the full-length SLC35D3 and the SLC35D3-NT bound with Myc-D1R. (**C**) Two heterozygous mutations were identified in two patients with MetS. WT: wild-type SLC35D3; MU: mutant SLC35D3. In patient #1, a 3-bp deletion (c.1209_1211delGAA) as underlined led to an in-frame deletion of K404. In patient #2, a 3-bp insertion (c.601_602insCTG) as the arrow indicated led to an in-frame insertion of L201. (**D**) In HEK293T cells, wild-type SLC35D3 was partially colocalized with EEA1 (early endosome) and SEC61B (ER), without colocalization with LAMP3 (late endosome/lysosome). The ΔK404 and insL201 mutations were mislocalized mainly on late endosomes/lysosomes.

We then tested whether there is a physical interaction between SLC35D3 and D1R by co-immunoprecipitation. Indeed, we observed that Myc-SLC35D3 co-precipitated with Flag-D1R (Fig. 6A). Reciprocally, Myc-D1R co-precipitated with Flag-SLC35D3 (Fig. 6B). In addition, we found that the N-terminal portion of SLC35D3 (1–241aa) interacted with the C-terminal region of D1R (217- 446aa) (Figs. 6A, 6B).

Taken together, our results indicate that SLC35D3 is likely involved in the membrane trafficking of D1R on its ER exit, and that loss of SLC35D3 leads to the intracellular D1R retention mainly on ER, thus reducing the amount of plasma membrane D1R receptors and their signaling.

### Mutational screen of human *SLC35D3* in patients with metabolic syndrome

The above findings in *ros* mice prompted us to investigate whether there are mutations in the orthologous human *SLC35D3* gene in patients with MetS. We screened 363 Chinese Han patients with MetS and 217 unaffected individuals by sequencing the two exons and adjacent exon/intron boundaries together with 1 kb untranslated sequence upstream of the start codon of the *SLC35D3* gene.

Two variants of SLC35D3 leading to the frame-shift of the coding sequence were found in two unrelated patients. These variants were absent in the control group or the NCBI SNP database for the *SLC35D3* gene (Locus ID 340146, http://www.ncbi.nlm.nih.gov/SNP/). In patient #1 (Male, Age: 55, BMI: 26.1, waist circumference: 109 cm, blood pressure: 135/85 mmHg, TG: 4.23 mmol/L, Chol: 5.28 mmol/L, Gluc: 4.4 mmol/L), a heterozygous ΔK404 was identified (Fig. 6C). The mutated SLC35D3 showed the miscolocalization to LAMP3-positive late endosomes/lysosomes in transfected cells compared with WT protein (Fig. 6D), suggesting its subcellular localization has been altered. In patient #2 (Male, Age: 51, BMI: 27.1, waist circumference: 100 cm, blood pressure: 120/80 mmHg, TG: 2.52 mmol/L, Chol: 5.94 mmol/L, Gluc: 5.2 mmol/L), a heterozygous insL201 was identified (Fig. 6C). Similarly, the mutant insL201 colocalized with LAMP3, but not EEA1 or SEC61B (Fig. 6D), also suggesting that these mutations alter the subcellular localization of SLC35D3. The residues around L201 are conserved in human, chimpanzee, dog, mouse and rat. However, the residues around K404 are less conserved in these species. The mislocalization of these two variants (insL201 and ΔK404) implicates localization or sorting signals may lie on these mutational sites.

We did not find a second mutation in the *SLC35D3* gene in these two patients after excluding possible large deletions, suggesting that both patients are likely affected in the heterozygous state. Both patients were diagnosed as having MetS with central obesity according to the guidelines of International Diabetes Federation (IDF) [15] and central obesity in China [16]. Similarly, we observed moderate weight gain in heterozygous *ros^+/−^* mice at 5 months of age compared with WT littermates (WT: 29.4±0.38, n = 7; *ros+/−*: 30.9±0.24, n = 8; *P*<0.01). The more severe weight gain in homozygous *ros*−/− mice at the same age is suggestive that the *SLC35D3* mutation may have a gene dosage effect on D1R trafficking. It is unknown whether patients with homozygous or compound mutations may have more severe phenotypes. Unfortunately, we were not able to get access to the blood samples of the family members of these two patients, which precluded us to explore the penetrance of the mutations. This study suggests that mutant human SLC35D3 does not function properly in the ER exit of D1R, thus likely impairing the membrane trafficking of D1R and D1 signaling in the patients in a similar mechanism as revealed in *ros* mice.

### D1R agonist reduces body weight and reverses hyperlipidemia in *ros* mice

**Figure 7:**
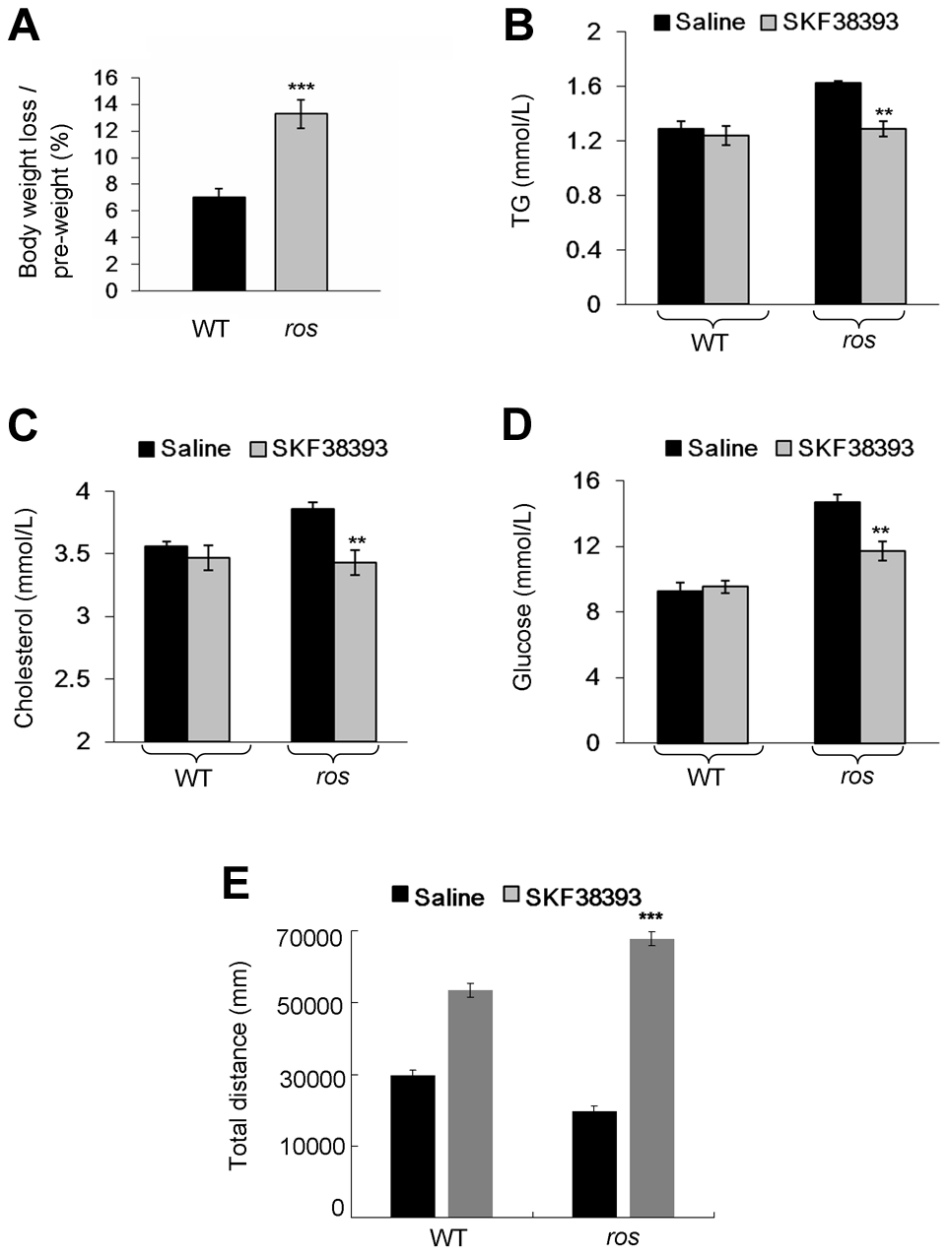
Effect of SKF38393 treatment on body weight loss, serum lipids and glucose, and activity of wild-type or *ros* mice. Mice at 24 weeks of age were used in this study. (**A**) Wild-type (WT) or *ros* mice (n = 9 per group) received a daily intraperitoneal injection of 20 mg/kg SKF38393 for 12 days and weighed on day 14. Body weights of saline-treated WT or *ros* mice had no significant changes, whereas body weights of SKF38393-treated WT mice were reduced approximately 7% (from 29.61±0.76 g to 27.5±0.6 g), and body weights of SKF38393-treated *ros* mice were reduced approximately 13% (from 38.56±0.79 g to 33.39±0.58 g). ***P<0.001. (**B–D**) Compared with saline-treated WT mice (n = 8), SKF38393-treated WT mice (n = 9) exhibit no significant changes of serum levels of triglycerides (TG), cholesterol (Chol) and blood glucose (Gluc). In SKF38393-treated *ros* mice, serum levels of Chol, TG and Gluc were significantly reduced compared to saline-treated *ros* littermates. SKF38393 vs. saline, Chol: 3.43±0.08 mmol/L (n = 9) vs. 3.86±0.05 mmol/L (n = 7); TG: 1.29 ± 0.06 mmol/L (n = 8) vs. 1.63±0.01 mmol/L (n = 7); Gluc: 11.7±0.57 mmol/L (n = 9) vs. 14.67±0.46 mmol/L (n = 7). **P<0.01. (**E**) Total distances traveled during the period of 30 min to 60 min after SKF38393 treatment were increased in both WT and *ros* mice. However, the distance traveled in *ros* mice was greater than that in WT (***P<0.001).

To test whether the pathological phenotype in obese *ros* mice is reversible by the treatment with a D1R agonist, adult male mice received a daily intraperitoneal injection of D1 receptor agonist SKF38393. Following the 12-day treatment period, we observed that body weight loss of *ros* mice (13%) was significantly higher than that of the wild-type (7%). In contrast, body weight changes of saline-treated wild-type mice or *ros* mice were not significant (Fig. 7A). Treatment with SKF38393 did not change the levels of serum lipids and glucose compared with saline-treatment in wild-type mice. Strikingly, serum cholesterol and triglycerides levels were significantly decreased for SKF38393-treated *ros* mice to levels that were similar to those of wild-type mice. Blood glucose levels in *ros* mice were significantly reduced after the treatment of SKF38393 (Figs. 7B–7D). Thus, administration of SKF38393 caused body weight loss and rescued the hyperlipidemia in *ros* mice. In addition, physical activity was increased significantly after SKF38393 treatment in *ros* mice compared with WT (Fig. 7E). These results suggest that impaired D1R signaling could be reversible by D1R agonists in *ros* mice and likely in patients with MetS who carry SLC35D3 mutations.

## Discussion

Obesity is caused by perturbations of the balance between energy intake and energy expenditure, which in turn is regulated by a complex physiological system that requires the coordination of several peripheral and central signals in the brain [17]–[19]. Dopaminergic signaling pathways are involved in the regulation of food intake and energy expenditure, including the mesolimbic pathway in food reward, the mesohypothalamic pathway in satiety and the nigrostriatal pathway in energy expenditure [20]–[22]. Both the D1 and D2 dopamine (DA) receptors act synergistically in the regulation of the basal ganglia function in the striatal MSNs [23], [24]. Dysregulation of DA signaling has been previously implicated in the development of obesity [25]. However, the precise mechanism by which DA receptors regulate energy balance is still unclear [26]. Positron emission tomography revealed that striatal D2 receptor availability is lower in obese humans compared to lean individuals [25], [27], but to date no human imaging studies have assessed the involvement of D1 receptors in obesity.

Reduction of DA receptors on the cell surface could result from 1) increased internalization, 2) reduced reinsertion to the plasma membrane due to increased degradation, and 3) reduced trafficking or expression *ab initio*. Previous extensive studies have focused on understanding the internalization of DA receptors following agonist occupancy, including agonist-elicited receptor desensitization, endocytosis, and resensitization or degradation [28]–[31]. Unlike D2R, which is generally trafficked to the lysosomes for degradation [32], endocytosed D1R is recycled back to the plasma membrane [14], [33]. However, mechanistic studies of the trafficking of D1R to the cell surface are limited [34], and its relevance of this trafficking to metabolic disorders has not been reported.

Transit out of the ER has been shown to be a critical control point and rate-limiting step in the expression of D1 receptors at the cell surface [35], [36]. A number of DA receptor-interacting proteins have been identified [37]. One ER protein, DRiP78, acts as a chaperon for D1 receptor trafficking [35]. Similarly, our data have shown that SLC35D3 is localized to the ER and endosomes, where it interacts with D1R. Loss of SLC35D3 in *ros* mice blocks the ER exit of D1R, thus leading to retention in the ER and reduced D1R distribution on the cell surface, thereby impairing D1R signaling. We have not completely excluded the possibility that D1R trafficking from early endosomes to plasma membrane is also blocked. Given that the ER-retained fraction of D1R accounts for the greatest proportion of reduced plasma membrane D1R (Figs. 4B and 5E), we speculate that SLC35D3 plays a major role in the ER exit of D1R. Likewise, in human patients, the mutant SLC35D3 (insL201 or Δ404K) is mistargeted to late endosomes/lysosomes and therefore is likely unable to function properly in the ER exit of D1R. Therefore, SLC35D3 is identified as a novel regulator of D1R membrane trafficking from ER.

The restricted expression of SLC35D3 in the brain and the absence of expression in other peripheral organs (except for the platelets) laid the foundation of our hypothesis that the MetS phenotype in *ros* mice is attributable to lesions in the central nervous system. Since D2R distribution (Fig. S2) is unaffected, and SLC35D3 was not expressed in D2 neurons [12], the impaired D1R signaling perturbs the D1R/D2R balance, which likely led to the reduced movement and energy expenditure due to the dysfunction of the basal ganglion DA loop.

No apparent obesity phenotypes have been documented in several *D1r*-knockout (KO) mouse lines as listed in the MGI database (MGI:99578, http://www.informatics.jax.org/). However, reduced spontaneous locomotor activity was reported in a line of *D1r*-KO mutants [38]. Although the number of plasma membrane D1R in *ros* mice is about 65% (34%/51.7%) of the WT mice (Fig. 4C), the *ros* mutant does not mimic the *D1r*+/− mice as the total number of D1R is unchanged but redistributed mainly from the plasma membrane to ER. The other significant difference is that SLC35D3 is selectively expressed in striatal D1R-expressing neurons which may manifest specific effects related to D1R reduction. The *D1r*-KO mice in contrast may have additional defects given the wider expression of D1R in both neuronal and non-neuronal tissues. In fact the *D1r*-KO mice showed postnatal growth retardation [39]. Thus, complex multiple interacting effects may preclude the development of obesity in the *D1r*+/− or *D1r*−/− mice. In other words, the *ros* mutant mouse mimics a D1-neuron specific knockdown of plasma membrane D1R, rather than mimicking the conventional *D1r*−/− or *D1r*+/− mouse.

In contrast to the *ob/ob* mice which develop obesity from the age of weaning [40], the *ros* mice exhibited progressive weight gain starting from 2 months of age. The delayed weight gain in *ros* mice with late-onset obesity is still a mystery given that the indicated protein is present in early stages after birth (Fig. S1). In addition, the two patients with *SLC35D3* mutations were diagnosed with adult central obesity, which also suggests a late-onset obesity phenotype in humans.

Our studies have elucidated the underlying genetic entity of a long-standing unresolved linked locus near the marker D6S1009. Both mouse and human mutations of the *SLC35D3* gene are associated with MetS, suggesting that *SLC35D3* is a novel candidate gene for MetS. Considering that obesity affects 10∼25% of the European population and nearly one third of the US population [41], a mutational screen of *SLC35D3* in the obese population would be cost-effective as a precursor to potential D1R agonist treatment.

Obese children receiving D1R agonist treatment reverse weight gain [42]. Similarly, administration of D1R agonist reversed most of the phenotype of MetS in *ros* mice. This effect may be caused by the stimulation of the residual D1R on the plasma membrane of *ros* striatum, or redirection of the ER-retained D1R to plasma membrane for its signaling. In addition, the reversible phenotypes upon D1R agonist treatment suggest that the reduced D1R numbers on the plasma membrane could be the primary cause of MetS in the *ros* mutant mice, although we have not excluded the effects on the substantia nigra and olfactory bulb where SLC35D3 are also expressed (Fig. 3A). SLC35D3 deficiency caused obesity primarily via effects on physical activity levels, supporting that genetic factors could be a component of low physical activity [43]. As reduced physical activity is the primary consequence of impaired D1R signaling, encouraging elevations in physical activity in these patients might be an alternative way to prevent or alleviate their symptoms of obesity [44].

## Materials and Methods

### Mice

The *ros* mutant (*ros−/−*) [10] and control C3H/HeSnJ mice (wild-type, WT) were originally obtained from Dr. Richard T. Swank's laboratory and bred in the animal facility of the Institute of Genetics and Developmental Biology (IGDB), Chinese Academy of Sciences. To ensure the genotypes of *ros−/−* and wild-type, we developed a PCR method of genotyping based on the nature of the insertional mutation in the *Slc35d3* gene [10]. For the locomotion tests, only male mice of each genotype at 12 weeks old were selected to control for potentially confounding hormonal effects during the estrous cycle in females. Mice were housed in a room with a 12-hr light/dark cycle (lights on at 7:30 a.m. and off at 7:30 p.m.) with access to food and water *ad libitum*. For other phenotypic analyses, males at 24 weeks of age were used except for those specified in figure legends or materials and methods.

### Study subjects

We recruited 363 unrelated Chinese Han patients with MetS from The Affiliated Hospital of Qingdao University Medical College and The Affiliated Children's Hospital of Nanjing Medical University, and 217 unaffected individuals from Beijing Tongren Hospital of Capital Medical University. The patients were diagnosed as MetS according to the guidelines of International Diabetes Federation (IDF) [15] and central obesity in China [16]. In brief, MetS is diagnosed as abdominal obesity (or central obesity) with any two of the following parameters, 1) TG>1.7 mmol/L, 2) HDL<1.03 mmol/L (male) or <1.29 mmol/L (female), 3) blood pressure >130/85 mmHg, 4) fasting plasma glucose >5.6 mmol/L. For the diagnosis of abdominal obesity in Chinese population, we choose either 1) BMI >28 as general obesity or 2) BMI between 24 to 28 as overweight, and waist circumference >90 cm (male) or >85 cm (female). Eight mililiter peripheral blood samples were collected from all subjects participating in this study. We designed primers for amplifying the two exons and about 1 kb upstream of the human *SLC35D3* gene. Amplified PCR products were subjected to direct sequencing by an ABI PRISM 3700 automated sequencer (Applied Biosystems, Foster City, CA).

### Measurements of obesity features and metabolites in mice

Growth curves for males were obtained by measuring body weight once a month from 4 to 24 weeks of age. For determination of body length, mice were anesthetized and fully extended to measure the naso-anal distance. Epididymal and perirenal fat pads were harvested from male mice and weighed. Blood was collected by cardiac puncture after an overnight fast for measuring blood glucose, serum cholesterol and triglycerides by colorimetric kit assays (Leadman, Beijing, China) and analyzed using an automatic biochemical analyzer (Hitachi, Tokyo, Japan). Insulin was measured by a rat/mouse insulin enzyme-linked immunoassay (ELISA) Kit on non-fasted mice (Millipore, Bedford, MA, USA). For the insulin tolerance test (ITT) and glucose tolerance test (GTT), fasting plasma glucose levels were measured (16 hours fast, blood taken from the tail vein) using a glucosimeter (Teromo, Japan). Then insulin (Roche Diagnostics, Switzerland) was injected intraperitoneally (1 U/kg) and blood glucose was measured again at 15, 30, 60, 90 and 120 min post injection. Alternatively, D-glucose (Sigma-Aldrich, St. Louis, Missouri, USA) was injected intraperitoneally (2 g/kg body weight) and blood glucose was measured again at 15, 30, 60 and 120 min post injection.

### Spontaneous locomotor activity

Mice were pre-exposed to the chamber before testing to allow environmental habituation, and activity was monitored under indirect dim light and sound-attenuated conditions. A single mouse was placed in a chamber (40 cm length×40 cm width×45 cm height) for 30 min. Total distance traveled, average velocity and total movement duration measured spontaneous activity. All these parameters were measured by JLBehv software (JLGY, Shanghai, China). Behavioral testing was performed between 8:00 and 12:00 a.m.

### Food intake

Before measurement of daily food intake, mice fed *ad libitum* were individually housed for 3 days to allow environmental habituation. Food was measured at 3:00 p.m. each day for 7 consecutive days.

### VO_2_ and VCO_2_ measurements

Mice aged 8 to 12 weeks (prior to development of obesity) were measured using an indirect calorimetry system (Oxymax, Ohio, USA). Oxygen consumption, CO_2_ production and physical activity (beam breaks) were recorded at 30-min intervals for 5 consecutive days (48 times a day). Volume of oxygen consumption (VO_2_) and carbon dioxide production (VCO_2_) were measured using electrochemical and spectrophotometric sensors respectively. Oxygen consumption data were converted to energy expenditure (Watts) using the measured RQ values using procedures outlined in Arch et al [45]. To report the total energy expenditure we averaged the 48 measurements collected each day across days 2 to 5, allowing the animals to acclimate during the first 24 h in the chambers [13]. We recorded simultaneously the physical activity levels of the mice. Typical temporal patterns of oxygen consumption and physical activity are shown in Figs. S2A and S2B respectively. To establish the resting metabolic rate we used two different strategies. First we summarized all the half hourly oxygen consumption data in a histogram and then calculated the mean of the lowest 5% of values. Typical histogram for the data shown in Fig. S2A is shown in Fig. S2C. A second method was to use the regression approach outlined by Nonogaki et al [46]. This involved plotting the time matched data for VO_2_ and physical activity levels (Fig. S2D) and then evaluating the resting metabolic rate from the intercept of a fitted linear regression model. The relationship between total metabolic rate and body weight for each genotype is shown in Fig. 2E, and that for the two resting metabolic rate approaches in Figs. 2F and 2G respectively. Following derivation of the total and resting rates of metabolism we corrected for the potentially confounding effects of body weight (Figs. 2E–2G) as recommended in Arch et al [45] and Tschop et al [13].

### Treatment of D1 receptor agonists on mice

Twenty-four to 25-week-old male mice of each genotype received a daily intraperitoneal injection of dopamine D1 receptor SKF38393 (20 mg/kg) (Sigma-Aldrich) for a period of 12 days. Saline-treated mice served as controls. Body weight in all mice was measured on the fourteenth day. Blood glucose, serum cholesterol and triglycerides, and locomotor activities were measured as described above.

### cAMP ELISA measurement

The dorsal striatum of mice was dissected as above and homogenized in Buffer A (10 mM Tris pH 7.4, 1 mM EDTA, 30 µM leupeptin, 1 µM pepstatin A) with 10% sucrose. Membranes were isolated by centrifugation (65 min at 100,000 g) onto a cushion of Buffer A with 44.5% (w/v) sucrose. The membranes at the interface were transferred to a new tube and washed twice with Buffer A and collected by centrifugation (30 min at 100,000 g). Protein concentrations were determined with Protein Assay (Bio-Rad, Hercules, CA, USA). Adenylyl cyclase activity was determined by incubating membrane protein (20 µg) at 30°C for 10 min in 0.1 ml of buffer containing 10 mM imidazole (pH 7.4), 0.2 mM EGTA, 0.5 mM MgCl_2_, 0.5 mM DTT, 0.1 mM ATP, 0.5 mM IBMX, and 10 µM D1 receptor agonists SKF82958 (Sigma-Aldrich). Reactions were terminated by placing the tubes into boiling water for 2 min. The cAMP concentrations were measured using the Direct cAMP Enzyme-linked Immunoassay Kit (Sigma-Aldrich) following the manufacturer's instructions. Optical density was measured at 405 nm by a microplate reader (Bio-Rad).

### Antibodies

The polyclonal rabbit anti-mouse SLC35D3 antiserum (1∶1000) for immunoblotting (WB) was prepared using the purified C-terminal 322–422aa peptide as an antigen. The monoclonal mouse anti-GFP (1∶1000) and polyclonal rabbit anti-Myc (1∶1000) were obtained from Santa Cruz Biotechnology (Santa Cruz, CA, USA). Monoclonal mouse anti-Flag antibody (Sigma-Aldrich) was used for WB (1∶7000) and immunocytochemical (ICC) analysis (1∶3000). Mouse monoclonal antibody against calnexin (1∶200) for ICC was purchased from Abcam (Cambridge Science Park, Cambridge, UK). Mouse monoclonal antibody against D1 receptor (Chemicon, Temecula, CA, USA) was used for WB (1∶600) and ICC (1∶300). Polyclonal rabbit anti-D2 receptor (Millipore) was used for WB (1∶2000). Mouse polyclonal antibody against GM130 for ICC (1∶500) was a kind gift from Dr. S. Bao (IGDB, CAS, China). Polyclonal rabbit anti-LAMP3 (Chemicon) was used for ICC (1∶200). Rabbit polyclonal anti-SEC61B antibody was purchased from Millipore and used for ICC (1∶600). Mouse monoclonal anti-EEA1 was from BD Biosciences (Franklin Lakes, NJ, USA) for ICC (1∶500). Rabbit polyclonal anto-GluR1 was from Millipore for WB (1∶1000). Goat polyclonal anti-BIP was from Santa Cruz Biotechnology for WB (1∶400). Mouse monoclonal anti-β-actin (Sigma-Aldrich) was used for WB (1∶10000). Alexa Fluor 594-conjugated donkey anti-mouse and donkey anti-rabbit IgG (H+L) were purchased from Molecular Probes (Invitrogen, Carlsbad, CA, USA).

### Western blotting

Cell lysates, immunoprecipitates or tissue lysates were combined with loading buffer and subjected to 8–12% SDS polyacrylamide gel electrophoresis (SDS-PAGE). Proteins were blotted onto polyvinylidene difluoride membranes in phosphate buffer with 0.1% Tween-20 (PBST), blocked for 1 h in 5% non-fat dry milk/PBST and probed for 2 h with primary antibodies at room temperature. The membranes were rinsed three times (10 min each) with PBST prior to incubation with appropriate peroxidase-conjugated secondary antibodies (Santa Cruz Biotechnology) and developed with enhanced chemiluminescence (Amersham Biosciences, Piscataway, NJ, USA).

### Constructs and co-immunoprecipitation

We prepared constructs for co-immunoprecipitation and immunofluorescence experiments. Mouse entire coding regions of wild-type *Slc35d3* (RefSeq, NM_029529, http://www.ncbi.nlm.nih.gov/refseq/), *D1r* (RefSeq, NM_010076), *D2r* (RefSeq, NM_010077), and human *SLC35D3* (RefSeq, NM_001008783) were subcloned into the pEGFP-C2 or -N2 vector (with GFP-tag), pCMV-tag2B vector (with Flag-tag) and pCMV-tag3B vector (with Myc-tag) as specified in the figures. The fragments of mouse SLC35D3 and mouse D1R specified in figure legends were generated by subcloning. The mutant human *SLC35D3* constructs (ΔK404 and insL201) were generated by site-directed mutagenesis (Takara, Japan) using human wild-type *SLC35D3* construct.

Transfected HEK-293T cells grew to confluency on 6-well plates. Cells were harvested and lysed in 50 mM Tris-HCl (pH 7.4), 150 mM NaCl, 1 mM EDTA, 1% Triton X-100 and protease inhibitors. Cell lysates were centrifuged at 18,000 g for 10 min, and the supernatant was collected and recentrifuged. The supernatant was incubated overnight with 3 µg mouse monoclonal anti-FLAG M2 antibody (Sigma-Aldrich) and washed 6 times with ice-cold wash buffer (50 mM Tris-HCl, pH 7.4, 150 mM NaCl). The samples were eluted with elution buffer (5 µg/µl 3× FLAG peptide) and subjected to SDS-PAGE and Western blotting with anti-Myc or anti-Flag antibody as described above.

### Histological and immunohistochemical staining

Mice were perfused through the heart with 4% paraformaldehyde in 0.1 M phosphate buffer (pH 7.4) under deep pentobarbital anesthesia. The brains were removed, and 20 µm frozen sections in the coronal plane were prepared for hematoxylin and eosin (H–E) or immunohistochemical staining (IHC). The H–E staining followed routine procedures. A standard H–E staining protocol was applied to sections of adipose tissue, liver, pancreas and skeletal muscle. For IHC, the endogenous peroxidase activity was blocked by treatment with 0.3% hydrogen peroxide in methanol, sections were blocked with 0.01 M PBS containing 10% goat serum and were then incubated overnight at 4°C with the mouse monoclonal antibody against D1R (1∶400). Following 0.01 M PBS rinses, sections were incubated in a biotinylated secondary antibody (Zhongshan Goldenbridge, Beijing, China) for an hour at room temperature, treated for another hour at room temperature with peroxidase-ligated streptavidin. The results were captured by a TS100 microscope (Nikon, Tokyo, Japan).

### Immunofluorescence confocal imaging

HEK293T cells transfected with EGFP-SLC35D3 (wild-type or mutant or other constructs as specified in the results or figure legends) were grown on glass cover slips in 24-well plates until 30–50% confluence. 18–20 hrs after transfection, they were fixed with freshly prepared 4% paraformaldehyde for 10 min. Cells were washed 3 times with 0.01 M phosphate buffer (pH 7.4). The permeabilization of cells was carried out in the presence of 0.3% Triton X-100 in PBS for 10 min. After blocking in 0.01 M PBS containing 1% BSA for 1 hr at 37°C, fixed cells were incubated with various antibodies as indicated in the results overnight at 4°C. Cells were then washed 3 times in 0.01 M PBS containing 0.1% Triton X-100 before incubating with Alexa Fluor 594-conjugated secondary antibody at 1∶2000 dilution for 1 h at 37°C. Cells were then washed 3 times before glass cover slips were mounted. Images were acquired with an ×100 lens on a D-ECLIPSE-si confocal microscope (Nikon).

### Immuno-electronic microscopy examination

Mice were anesthetized with pentobarbital (0.1 g/kg, Sigma-Aldrich). Brains were separated and cut by a vibratome (DSK, model DTK-1000, Japan). The striatum was fixed with 2% paraformaldehyde, 2.5% glutaraldehyde and 0.1% tannic acid in 0.1 M natrium cacodylicum. Then sections were rinsed and postfixed with 1% osmium tetroxide for 30 min. After washing, the sections were dehydrated in an ascending series of dilution of acetone and impregnated in Epon 60°C, 24 hours. Ultrathin (70 nm) sections were collected on nickel grids, rinsed and incubated with mouse anti-D1R (1∶10) or rabbit anti-SEC61B (1∶50) in 1% BSA buffer overnight at 4°C, washed and incubated with 10 nm gold-anti-mouse IgG or 15 nm anti-rabbit IgG (1∶50). Sections were observed in JEM 2000 electron microscope (Japan). All the reagents were purchased from Electronic Microscope Science (EMS, Hatfield, PA, USA).

### OptiPrep gradient assay

The dissected striatum was immediately homogenized with 1 ml HB lysis buffer (250 mM sucrose, 20 mM Tris-HCl, pH 7.4, 1 mM EDTA). The sample was placed onto the top of an 11 ml continuous 5%–20% Optiprep (Axis-Shield, Norway) gradient in HB buffer. The gradient was centrifuged at 200,000 g (34100 rpm) for 14 hours in a Beckman SW41 rotor. Twenty-six fractions (400 µl each) were collected from the top using auto-collector (BioComp, USA). Equal aliquots from each fraction were analyzed for immunoblotting.

For the fractionation assay, the dissected striatum was immediately homogenized with 200 µl HB lysis buffer. 800 µl 20% Optiprep and 800 µl 5% Optiprep in HB buffer were placed into the tube constitutively. The tissue lysate was placed onto the top of the gradient. The sample was centrifuged at 28,000 rpm (TLS-55, Beckman, USA) for 14 hours. Eighteen fractions (100 µl each) were collected from the top. Based on the pilot Western assay, the fractions were combined as the 1–10th tube (mostly cytoplasm fraction) and the 11–18th tube (mostly plasma membrane fraction) respectively. Equal aliquots from each fraction were analyzed for immunoblotting.

### Data collection and analyses

cAMP ELISA was performed in duplicate and was repeated three or four times. The standard curves were generated using non-linear regression curve fitting. The specific protein bands on Western blots were scanned and analyzed using the software program NIH Image J. All data were obtained from at least three independent experiments. Data were expressed as mean ± SEM and statistical significance was tested by Student's *t*-test. Data from the calorimetry system was tested by ANCOVA. Distribution of gold-labeled particles in immuno-EM pictures were counted and statistical significance was tested by Chi-square test. Intensities of immunofluorescence in cultured cells were analyzed using NIH Image J.

### Ethics statements

All mouse procedures were approved by the Institutional Animal Care and Use Committee of IGDB (mouse protocol KYD2006-002). The study of human subjects was approved by the Bioethic Committee of IGDB, Chinese Academy of Sciences (IRB approval number, IGDB-2011-IRB-002). The study was conducted according to the Declaration of Helsinki Principles. Written informed consents were obtained from all subjects.

## Supporting Information

Figure S1 Expression of SLC35D3 in postnatal mouse striatum. Wild-type (WT) mouse striatum were dissected from mice at different postnatal ages (0.5, 1, 2, 3, 5, 6 months) and the lysates were applied to Western blotting analyses. The striatum from 6-month-old *ros* mice were used as a control. β-actin was a loading control. No apparent expression level changes were noted in the blots. Two independent studies were performed.
(TIF)

Figure S2 Analysis strategy for oxygen consumption and energy expenditure measurements. (**A**) Typical pattern of oxygen consumption measured at 30 min intervals over a 5 day test period. (**B**) Simultaneous measurements of physical activity to the measurements of oxygen consumption shown in (A). (**C**) Histogram of the half hourly measurements in (A) and the cut-off used to determine the minimal (or resting) metabolic rate. (**D**) Plot of oxygen consumption against physical activity levels for the data in (A) and (B), showing the fitted regression and the estimated resting metabolism.
(TIF)

Figure S3 D2R expression and distribution in striatum. (**A**) No significant change of total level of D2R in striatum was observed between wild-type (WT) and *ros* mutant (*P*>0.05). Quantification of the intensities was calculated from three independent experiments. (**B**) Striatum lysates were fractionated into plasma membrane (PM) and cytoplasm (cyto) containing intracellular organelles. Distribution of D2R on the plasma membrane was normal in the *ros* mice compared to the wild-type (WT).
(TIF)
