# Supplementary figures and images for: Mutation of SLC35D3 Causes Metabolic Syndrome by Impairing Dopamine Signaling in Striatal D1 Neurons
Source: PLoS Genet. 2014 Feb 13;10(2):e1004124. doi: 10.1371/journal.pgen.1004124 (PMC3923682; doi:10.1371/journal.pgen.1004124)

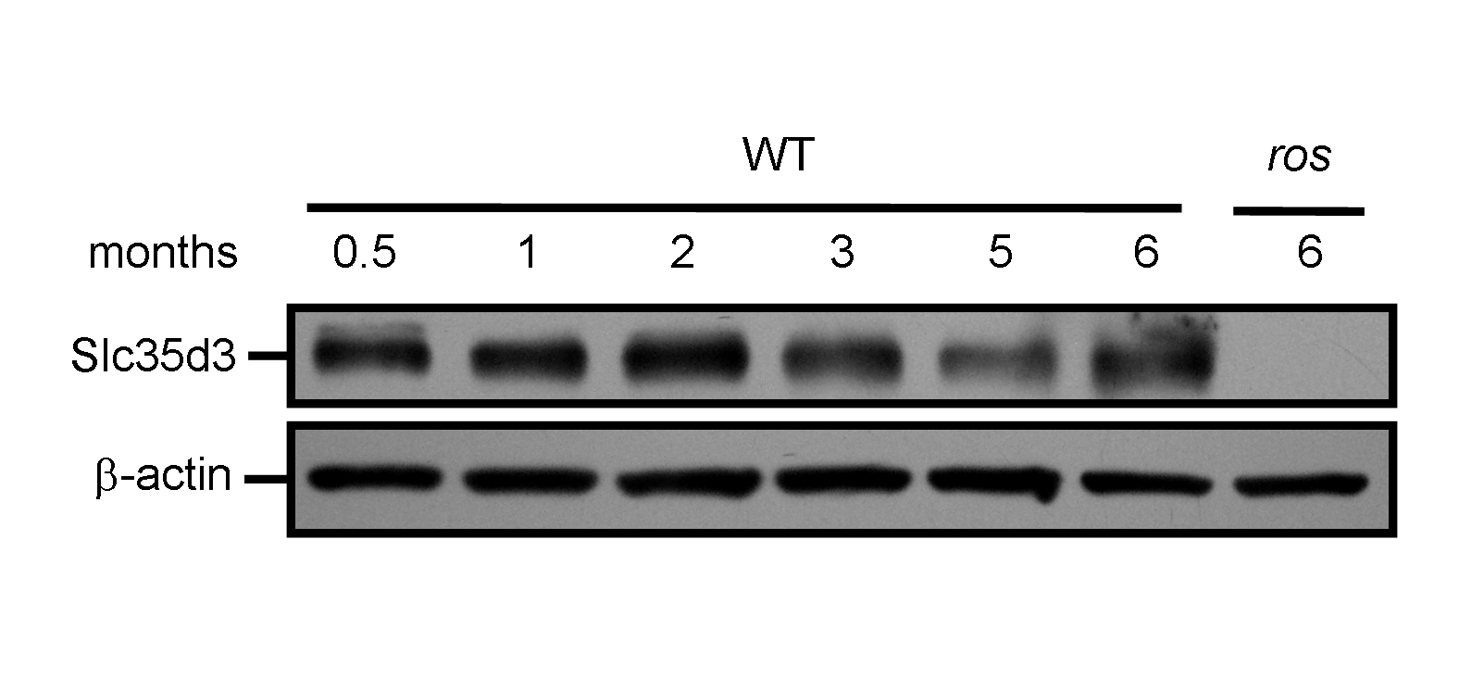

Supplement: Figure S1 — Expression of SLC35D3 in postnatal mouse striatum. Wild-type (WT) mouse striatum were dissected from mice at different postnatal ages (0.5, 1, 2, 3, 5, 6 months) and the lysates were applied to Western blotting analyses. The striatum from 6-month-old ros mice were used as a control. β-actin was a loading control. No apparent expression level changes were noted in the blots. Two independent studies were performed. (TIF) [file pgen.1004124.s001.tif]

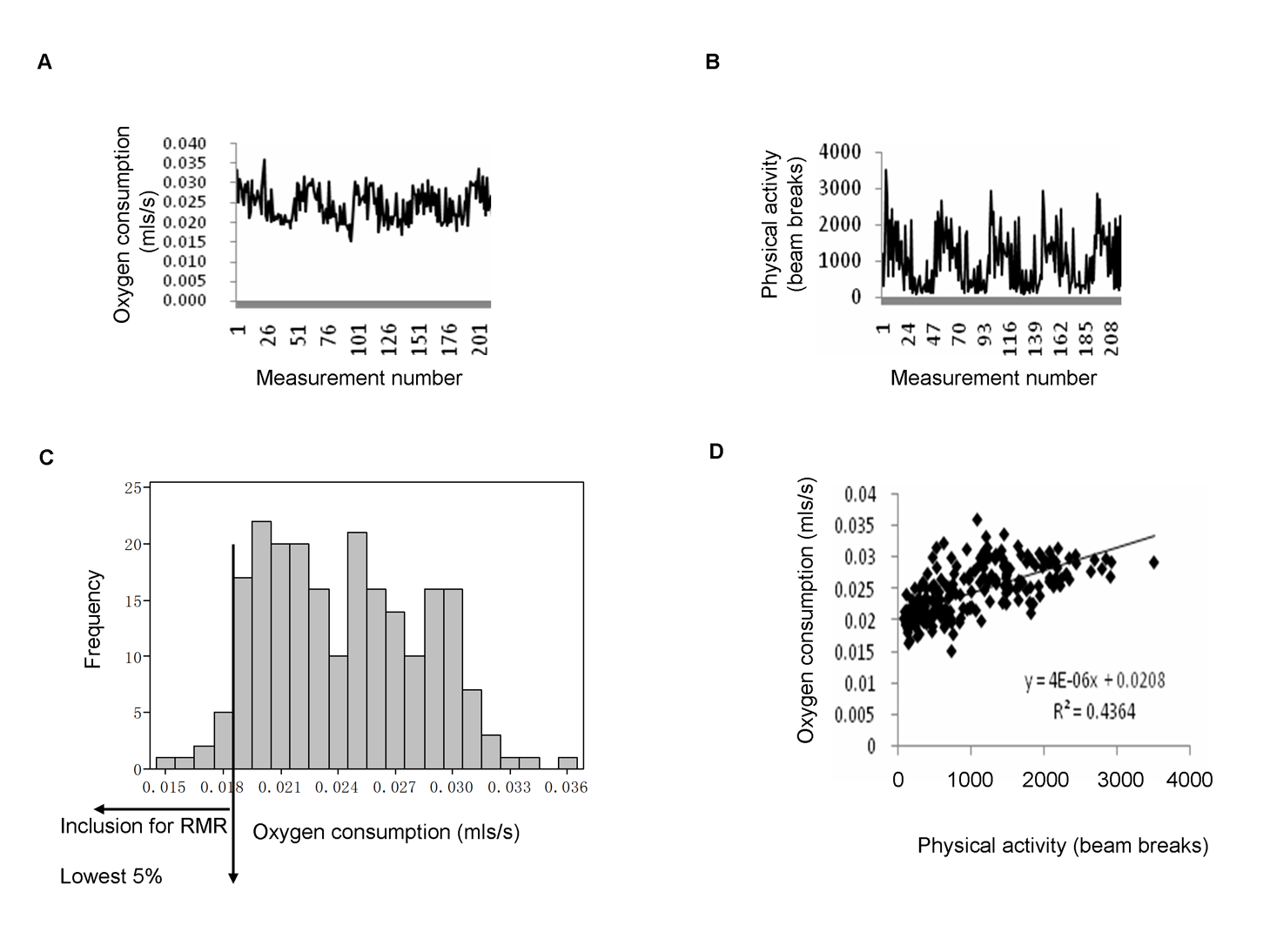

Supplement: Figure S2 — Analysis strategy for oxygen consumption and energy expenditure measurements. (A) Typical pattern of oxygen consumption measured at 30 min intervals over a 5 day test period. (B) Simultaneous measurements of physical activity to the measurements of oxygen consumption shown in (A). (C) Histogram of the half hourly measurements in (A) and the cut-off used to determine the minimal (or resting) metabolic rate. (D) Plot of oxygen consumption against physical activity levels for the data in (A) and (B), showing the fitted regression and the estimated resting metabolism. (TIF) [file pgen.1004124.s002.tif]

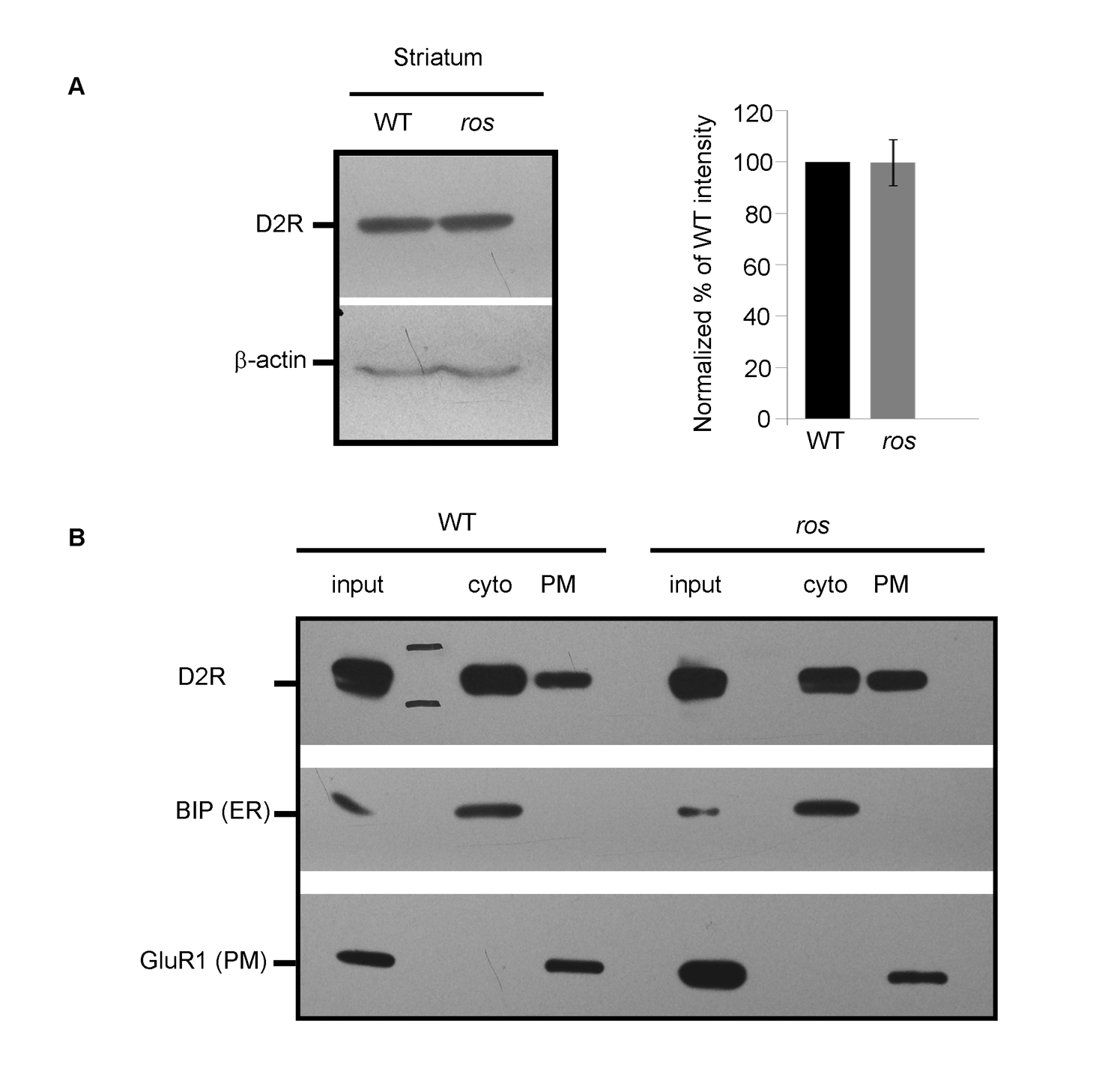

Supplement: Figure S3 — D2R expression and distribution in striatum. (A) No significant change of total level of D2R in striatum was observed between wild-type (WT) and ros mutant (P>0.05). Quantification of the intensities was calculated from three independent experiments. (B) Striatum lysates were fractionated into plasma membrane (PM) and cytoplasm (cyto) containing intracellular organelles. Distribution of D2R on the plasma membrane was normal in the ros mice compared to the wild-type (WT). (TIF) [file pgen.1004124.s003.tif]
